# Supplementary material for: Bioinformatic Analysis of Lytic Polysaccharide Monooxygenases Reveals the Pan-Families Occurrence of Intrinsically Disordered C-Terminal Extensions
Source: Biomolecules. 2021 Nov 4;11(11):1632. doi: 10.3390/biom11111632 (PMC8615602; doi:10.3390/biom11111632)
Supplement: Supplementary file 1 [file biomolecules-11-01632-s001.zip › biomolecules-1422186-supplementary.pdf]

biomolecules

Supplementary Information

Table S1. Number of LPMO sequences per family analyzed in this study.

AA Family	Before Curation	After Curation	Removed Sequences (%)
**AA9**	20,755	16,261	22%
**AA10**	6454	2872	55%
**AA11**	5261	4172	20%
**AA13**	662	475	28%
**AA14**	1657	1381	17%
**AA15**	648	517	20%
**AA16**	1770	1382	22%

Table S2. List of LPMO-dCTRs identified in secretomes and transcriptomes.

AA Family	Protein (UniProt) or Gene (GenBank)	Organism	Regulation	Growth On	Reference
AA9	EAA65609.1	*Aspergillus nidulans*	Transcription	Pectin	[64]
AA9	EAA64499.1/ Q5BAP2	*Aspergillus nidulans*	Transcription/ Secretion	Pectin, starch	[64,65]
AA9	EAA63617.1/ Q5B8T4	*Aspergillus nidulans*	Transcription/ Secretion	Cellulose, biomass sorghum, xyloglucan, starch	[64,65]
AA9	B2ARG6	*Podospora anserina*	Secretion	Sugar beet pulp	[66]
AA9	A0A1C9ZMC5	*Gloeophyllum trabeum*	Transcription	Highley's medium + glucose	[67]
AA9	BAV57614.1	*Gloeophyllum trabeum*	Transcription	Highley's medium + glucose	[67]
AA14	XP_007393138.1	*Phanerochaete carnosa*	Transcription	Aspen and spruce	[68]
AA9	XP_007400275	*Phanerochaete carnosa*	Transcription	Aspen and spruce	[68]

AA14	XP_007393767.1	*Phanerochaete carnosa*	Transcription	Aspen and spruce	[68]
AA9	G0SA07	*Chaetomium thermophilum*	Secretion	arabinose and microcrystalline cellulose	[69]
AA9	G0S4L0	*Chaetomium thermophilum*	Secretion	arabinose and microcrystalline cellulose	[69]
AA9	OSC96684.1	*Pycnoporus coccineus*	Transcription	Maltose, pine and aspen	[70]
AA9	OSC98651.1	*Pycnoporus coccineus*	Transcription	Maltose, pine and aspen	[70]
AA9	OSC98645.1	*Pycnoporus coccineus*	Transcription	Maltose, pine and aspen	[70]
AA9	OSC98630.1	*Pycnoporus coccineus*	Transcription	Maltose, pine and aspen	[70]
AA9	OSD06900.1	*Pycnoporus coccineus*	Transcription	Maltose, pine and aspen	[70]

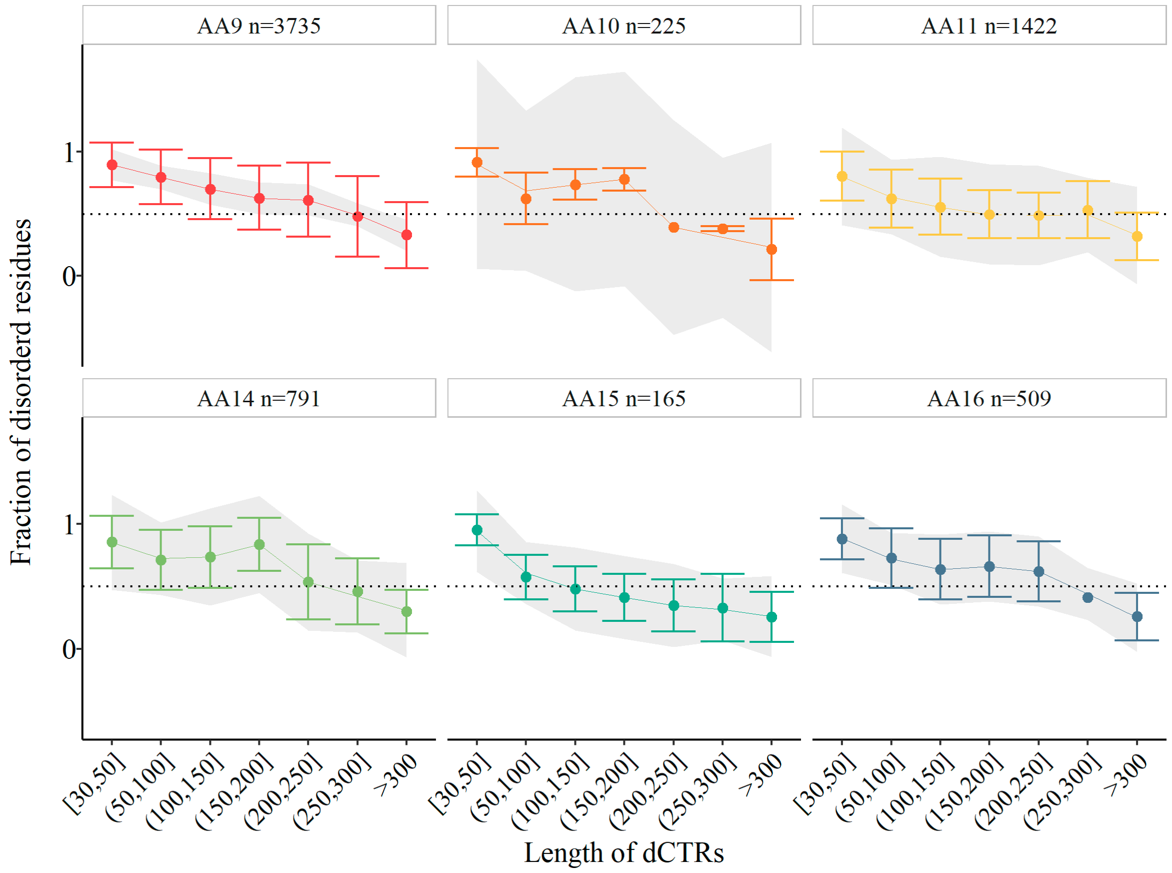

Figure S1. Length and disorder content of dCTRs. The graphs show the mean value of fraction of disordered residues of dCTRs with different lengths across LPMOs families. The error bars denote the standard deviation of the mean, the area in grey denotes the 95% confidence level interval. The dotted line represents the fraction of disorder residues equal to 0.5.

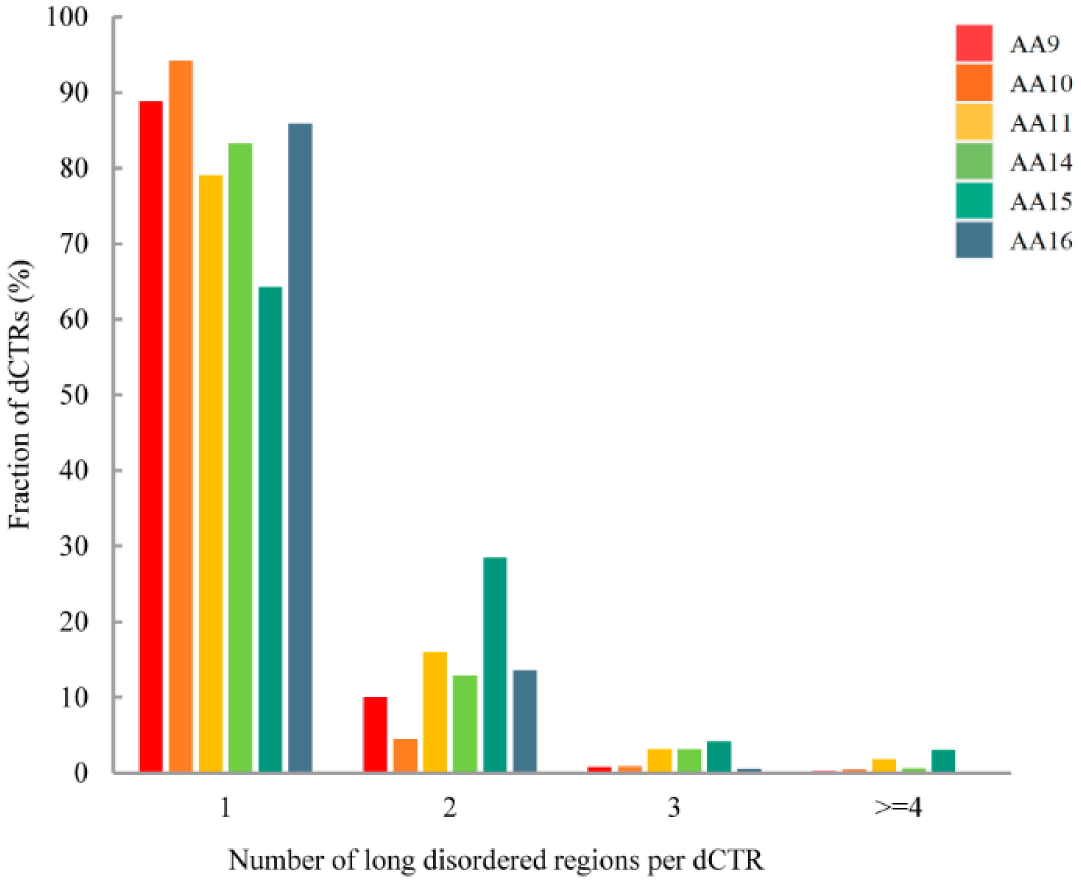

Figure S2. Frequency distribution of the number of long disordered regions (LDRs) per dCTR across the different LPMO families.

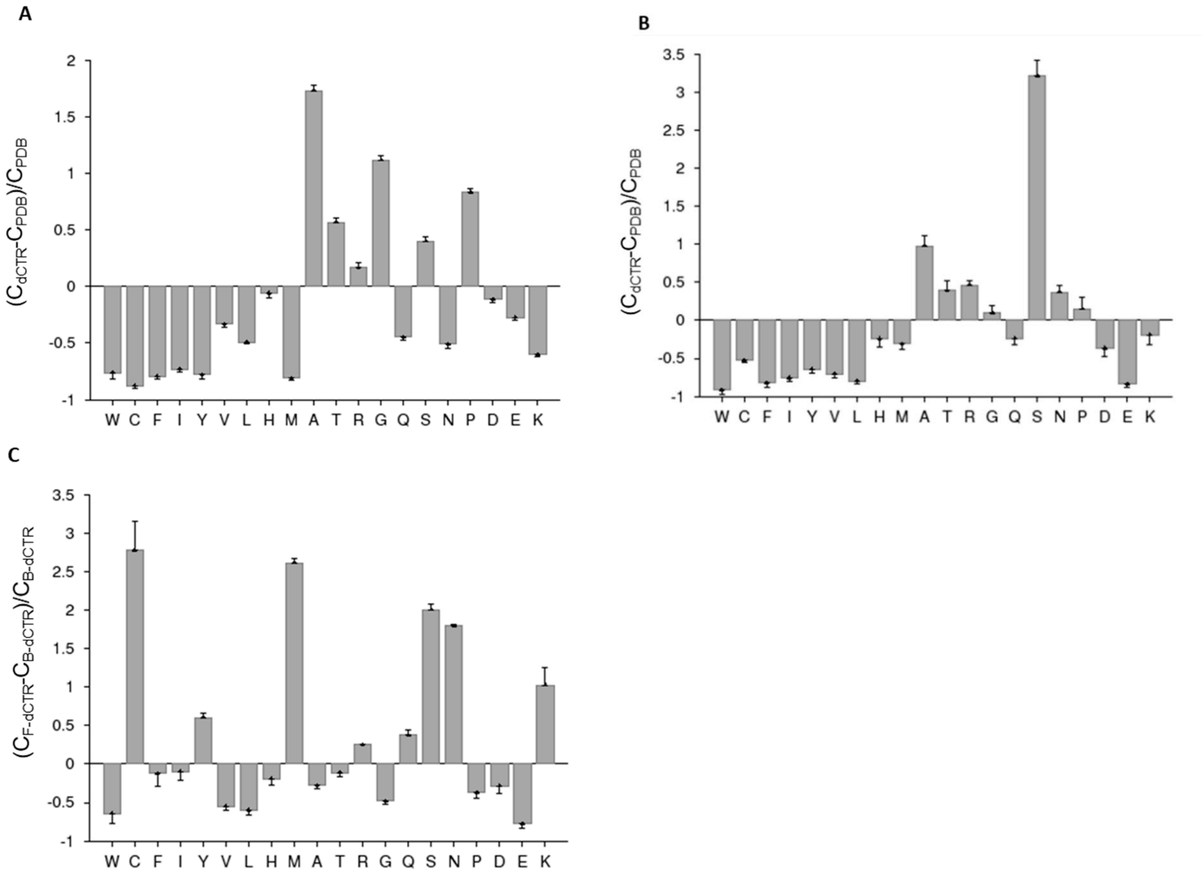

Figure S3. Compositional profile of dCTRs from AA10 LPMOs as generated by the Compositional Profiler tool (http://cprofiler.org/cgi-bin/profiler.cgi). (**A,B**) Compositional profile of dCTRs from bacterial (**A**) or fungal (**B**) AA10 with respect to the PDB database. In the two graphs, the fractional difference in the amino acid composition was evaluated as $(C_{dCTR}-C_{PDB})/C_{PDB}$, where C_{dCTR} is the content of the given amino acid in the ensemble of dCTRs under investigation, and C_{PDB} is the corresponding content in the PDB database. (**C**) Difference in amino acid composition between fungal and bacterial AA10 dCTRs. The latter was calculated as $(C_{F\text{-}dCTR}-C_{B\text{-}dCTR})/C_{B\text{-}dCTR}$, where $C_{F\text{-}dCTR}$ is the content of a given amino acid in the ensemble of dCTRs in fungal AA10 and $C_{B\text{-}dCTR}$ is the corresponding content in bacterial AA10 dCTRs.

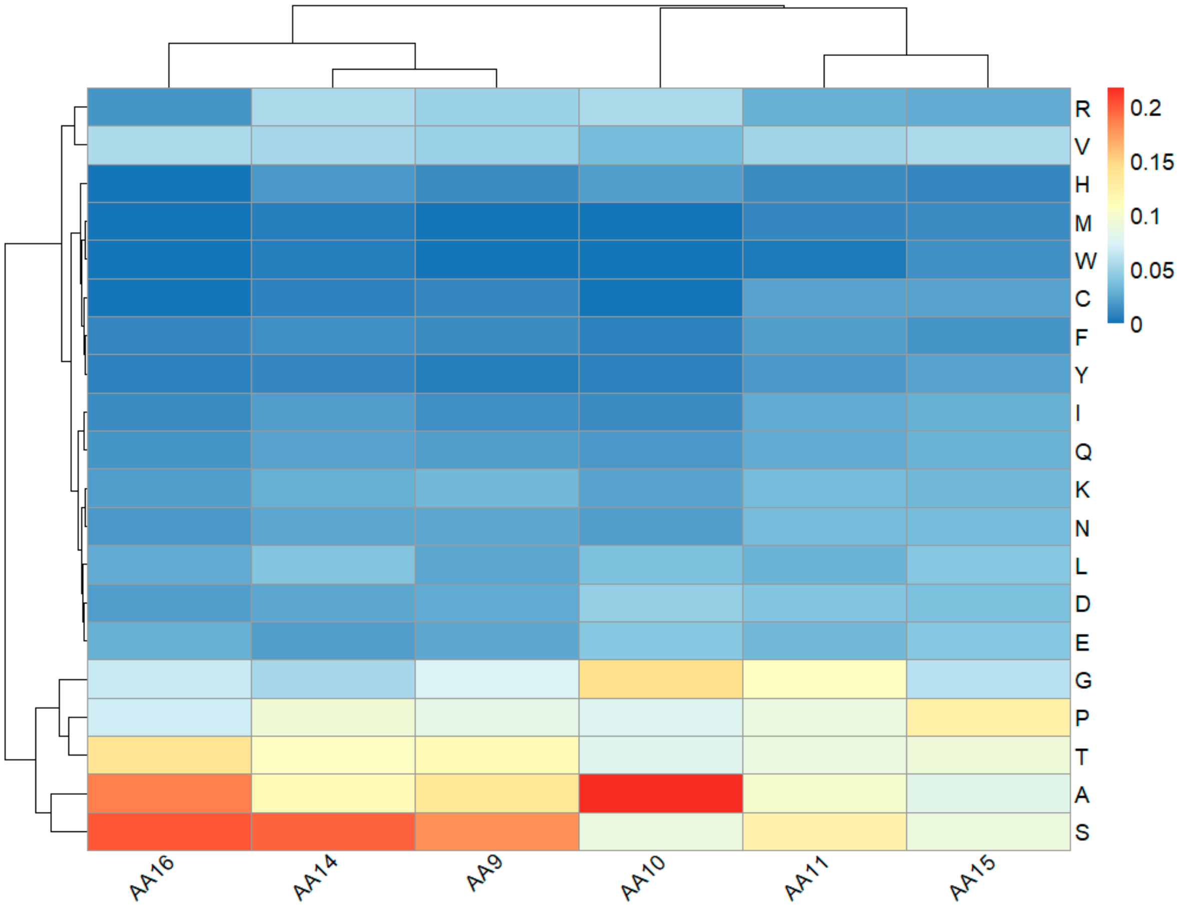

Figure S4. Amino acid composition of dCTRs across the different LPMO families. The heatmap shows the average amino acid content in the dCTRs of the different LPMO families. The color scale represents values ranging from 0 (blue) to 0.2 (red), where 0.2 indicates that an amino acid has a frequency of 20%, on average, in a given dCTR family. Clustering was performed by euclidean distance metric.

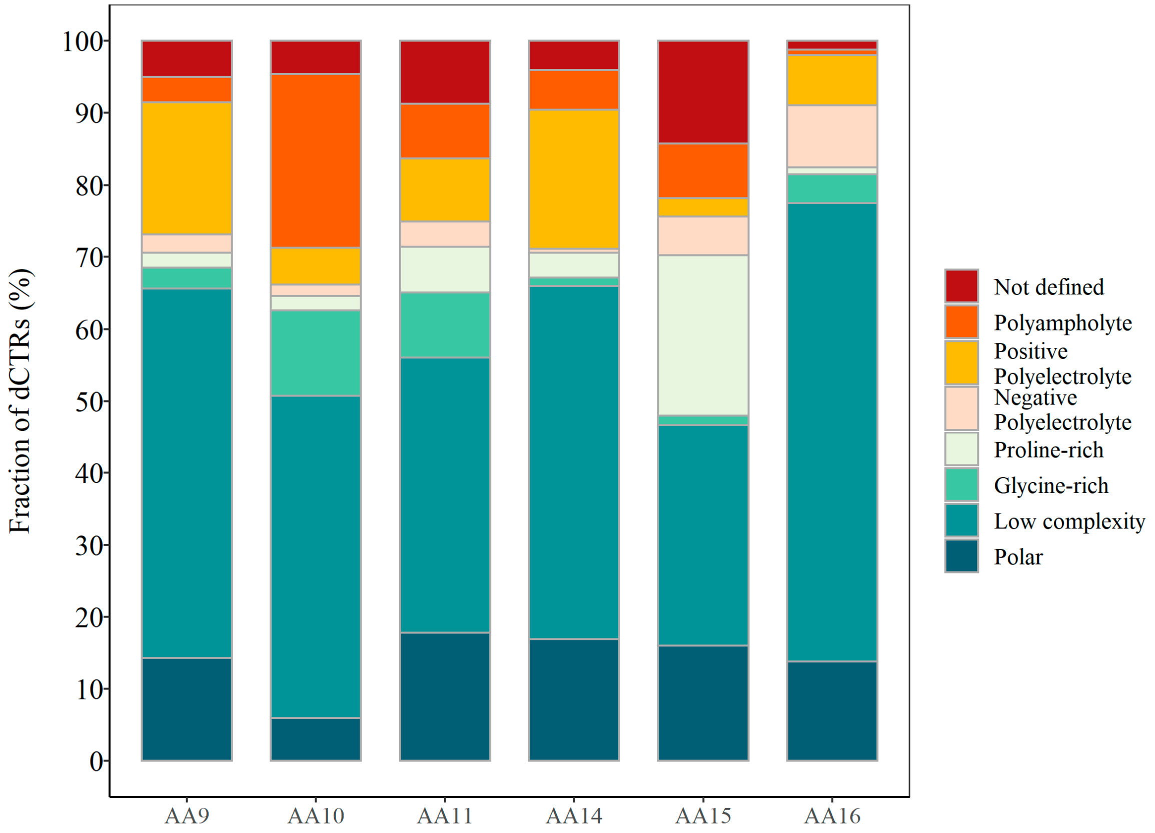

Figure S5. Occurrence of sequence features in dCTRs as predicted by MobiDB-lite 3.0. Each histogram divides each LPMO family based on seven sequence features, namely polyampholyte, positive polyelectrolyte, negative polyelectrolyte, Pro-rich, Gly-rich, low complexity and polar.

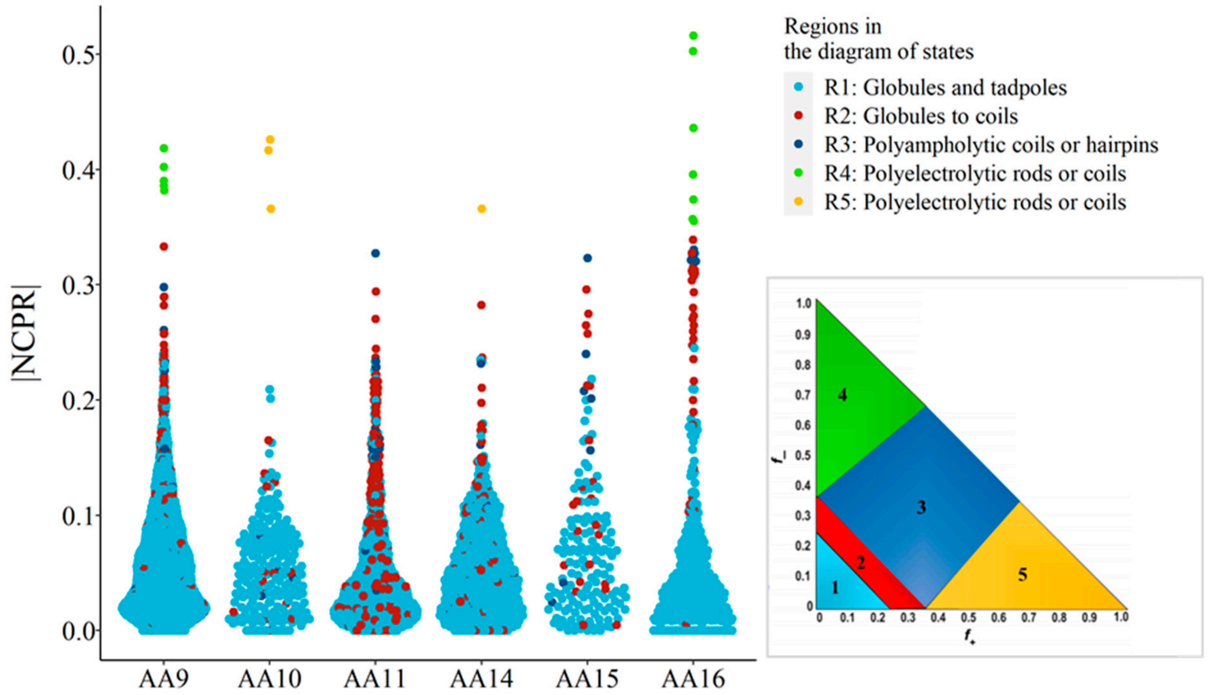

Figure S6. Distribution of absolute net charge per residue (NCPR) of dCTRs sequences across LPMO families. Each dot represents a sequence and the color of the dot is associated to the region the dCTR occupies in the diagram of states. The insert is a representation of the diagram of states. "f_-" is the fraction of negative residues, "f_+" is the fraction of positive residues.

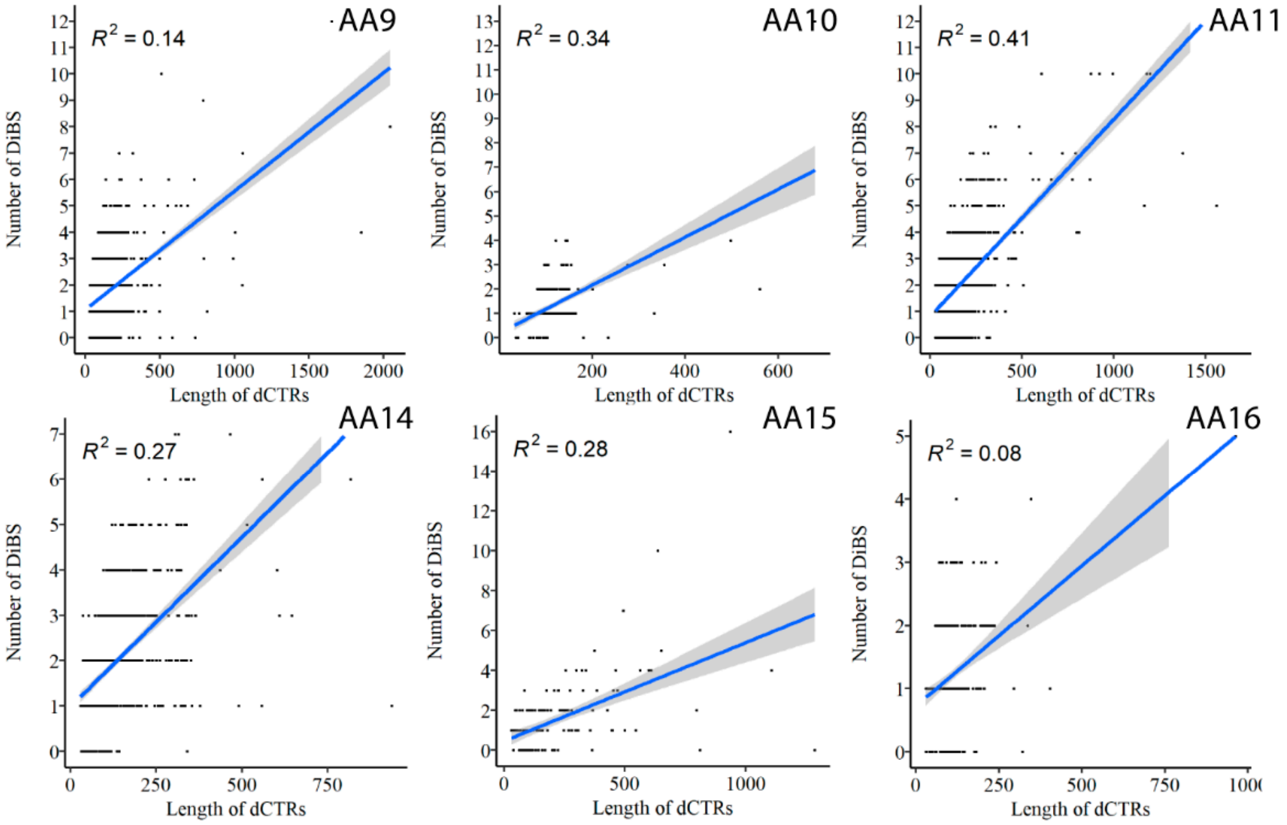

Figure S7. Scatter plot of the number of DiBSs as a function of the length of dCTRs across LPMO families. Each dot represents a sequence, the blue line is the regression line, and the area in grey denotes the 95% confidence level interval.

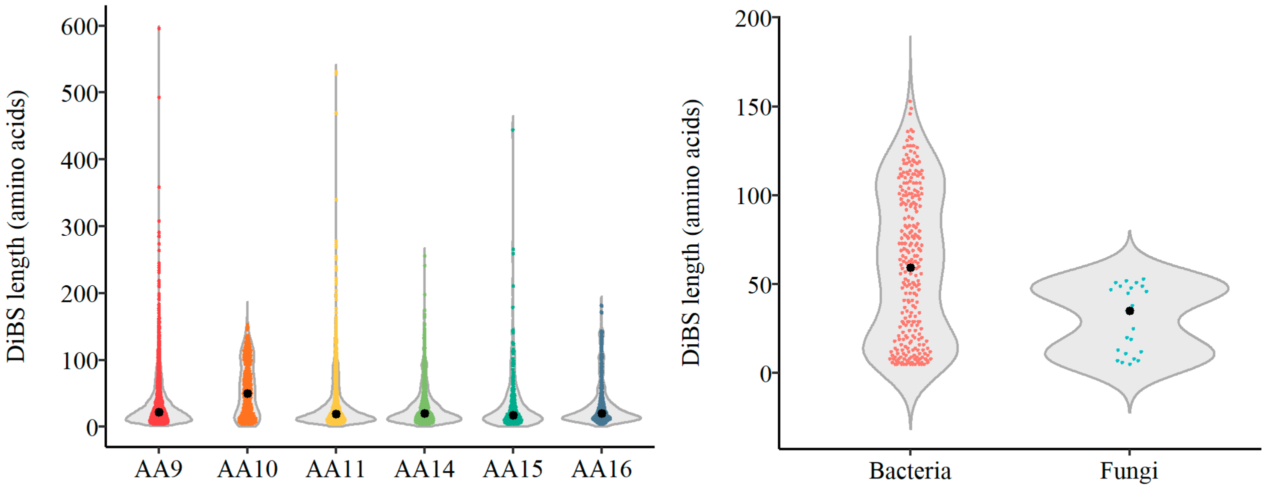

Figure S8. Length of DiBSs in dCTRs among LPMO families (**A**) and among bacterial and fungal dCTRs of the AA10 family (**B**). The black dot corresponds to the median.
